# Malaria infection in mosquitoes decreases the personal protection offered by permethrin-treated bednets

**DOI:** 10.1186/s13071-018-2846-0

**Published:** 2018-05-04

**Authors:** Kevin Thiévent, Lorenz Hofer, Elise Rapp, Mgeni Mohamed Tambwe, Sarah Moore, Jacob C. Koella

**Affiliations:** 1 Institute of Biology, University of Neuchâtel, Rue Emile-Argand 11, 2000 Neuchâtel, Switzerland; 2 Ifakara Health Institute, Intervention and Environmental Health and Ecological Sciences, P.O. Box 74, Bagamoyo, Tanzania; 3 Swiss Tropical & Public Health Institute, Socinstrasse 57, 4002 Basel, Switzerland

**Keywords:** Insecticide-treated bed nets (ITNs), Repellency, Personal and community protection, Behavioral manipulation, Malaria control

## Abstract

**Background:**

Insecticides targeting adult mosquitoes are the main way of controlling malaria. They work not only by killing mosquitoes, but also by repelling and irritating them. Indeed their repellent action gives valuable personal protection against biting mosquitoes. In the context of malaria control this personal protection is especially relevant when mosquitoes are infectious, whereas to protect the community we would prefer that the mosquitoes that are not yet infectious are killed (so, not repelled) by the insecticide. As the infectious stage of malaria parasites increases the motivation of mosquitoes to bite, we predicted that it would also change their behavioural response to insecticides.

**Results:**

With two systems, a laboratory isolate of the rodent malaria *Plasmodium berghei* infecting *Anopheles gambiae* and several isolates of *P. falciparum* obtained from schoolchildren in Tanzania that infected *Anopheles arabiensis*, we found that mosquitoes harbouring the infectious stage (the sporozoites) of the parasite were less repelled by permethrin-treated nets than uninfected ones.

**Conclusions:**

Our results suggest that, at least in the laboratory, malaria infection decreases the personal protection offered by insecticide-treated nets at the stage where the personal protection is most valuable. Further studies must investigate whether these results hold true in the field and whether the less effective personal protection can be balanced by increased community protection.

**Electronic supplementary material:**

The online version of this article (10.1186/s13071-018-2846-0) contains supplementary material, which is available to authorized users.

## Background

Indoor residual spraying (IRS) and insecticide-treated bednets (ITNs) are among the most commonly used and cost-effective control measures against malaria [1]. Indeed, these measures significantly reduced malaria transmission, for example, in sub-Saharan Africa [2]. Both protect against malaria by repelling mosquitoes and by killing mosquitoes that rest on the treated surface after having taken a blood meal [3–7]. ITNs give additional protection by creating a physical barrier that reduces human-mosquito contacts.

The repellent action of IRS and ITNs offers effective personal protection [8–11] by reducing the number of bites (infectious bites in particular) on users; their insecticidal action offers protection to the community, first, by killing mosquitoes before the malaria parasite has completed its development and become transmissible and secondly, by killing infectious mosquitoes [3]. A striking example of community protection is that a 75% coverage of ITNs in a rural community in Tanzania decreased the entomological inoculation rate of the people that did not use an ITN by a factor of 18 [12]. To some extent, the two levels of protection oppose each other: strongly repellent nets, which give strong personal protection, let only few mosquitoes contact the insecticide on the nets, thus killing few mosquitoes and giving weak community protection [3, 13–15].

To weaken this trade-off, we would want an ITN to repel mainly infectious mosquitoes, thus protecting users against being infected, but to repel other mosquitoes only weakly, thus maintaining community protection by lowering the number of mosquitoes that become infectious. Our prediction, however, was the opposite: as malaria changes the host-seeking behaviour of mosquitoes, its infectious stages would decrease repellency to give less rather than more personal protection against infection.

Indeed, infection with the infectious stage (sporozoites) increases the number of human-mosquito contacts in several ways. Sporozoite-infected mosquitoes have been found to be more attracted to human odours than uninfected ones [16, 17], though not all experiments show the same pattern [18]. Sporozoites also increase the mosquito’s persistence and biting rate [19–22] and increase the likelihood that the mosquitoes bite a second time to top up their blood meal [23]. These changes of behaviour probably evolved in order to increase the transmission of the parasite [24]. The general pattern of the behavioural changes - that sporozoite-infected mosquitoes are more motivated to bite than uninfected ones - may well have as a consequence that sporozoite-infected mosquitoes are less repelled by insecticides and therefore more likely to be killed by them.

The aim of this study was to test whether permethrin-treated bednets repel mosquitoes harbouring sporozoites less than uninfected mosquitoes (we chose to work with permethrin because it is one of the most used pyrethroids for insecticide-treated nets [25]). To do so, we performed two experiments in the laboratory that differed in the malaria parasite, the mosquito, and the way we measured the repellency of permethrin. In a first experiment with the rodent malaria *Plasmodium berghei* infecting *Anopheles gambiae*, mosquitoes chose between feeding through a permethrin-treated net or through an untreated net. In a second experiment with the human malaria *P. falciparum* infecting *An. arabiensis*, mosquitoes had to pass through a treated or untreated net if they wanted to reach the blood source. Note that, while for simplicity we use the term repellency in both experiments, we measure different behaviours. In the first, we measure *preference* of untreated nets, whereas in the second we measure the *motivation* to bite despite the presence of the insecticide. Note also that in both experiments, repellency refers to a combination of spatial repellency and contact irritancy [7], for our experimental design does not let us distinguish between the two.

## Methods

### *Plasmodium berghei *and *Anopheles gambiae*

#### Mosquito and parasite

We used the Kisumu colony of *An. gambiae* originating from western Kenya [26] and the mouse malaria *P. berghei* ANKA, modified to express the green fluorescent protein (GFP). Infected mice harbouring gametocytes were obtained from Volker Heussler (Institute of Cell Biology, University of Bern) and used to feed and infect the mosquitoes.

### *Mosquito rearing and infection*

We haphazardly selected larvae from the colony and reared them individually at 26 ± 1 °C in 12-well plates with a standard level of fish food (Tetramine) [26]: day of hatching of eggs, 0.04 mg per larva; 1 day after hatching, 0.06 mg; day 2, 0.08 mg; day 3, 0.16 mg; day 4, 0.32 mg; day 5 and later, 0.6 mg. Upon pupation, each mosquito was moved to a 120 ml plastic cup.

Three to four days after emergence, we moved the mosquitoes to an insectary maintained at 19 ± 1 °C and 70 ± 5 % of humidity for an efficient development of *P. berghei* in mosquitoes [27]. We haphazardly let females feed for 15 min on an infected or an uninfected mouse that had been anaesthetized. To replicate the infection-treatment, we used two infected and two uninfected mice, and let 100 mosquitoes feed on each one. Twenty-four hours after feeding, engorged mosquitoes were placed into 120 ml individual plastic cups; unfed mosquitoes were discarded. We gave the mosquitoes access to a cotton ball soaked with a 10% sugar solution up to 24 h before the behavioural test.

### *Measuring repellency*

**Fig. 1:**
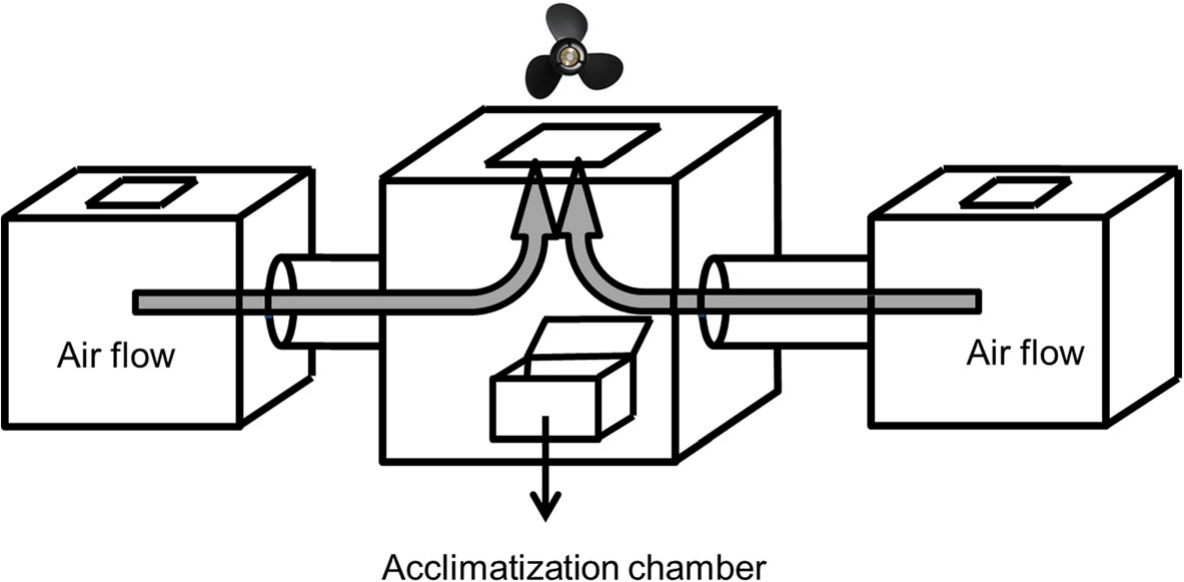
Two-way choice apparatus to test the effects of malaria infection on insecticide repulsion in the experiment with *P. berghei* infecting *An. gambiae*. The central cage (25 × 25 × 25 cm) was connected to two test cages (20 × 20 × 20 cm) with 15 cm length tubes (diameter 10 cm). The two small cages had a hole covered by the permethrin-treated net or the untreated net on the top. Mosquitoes were placed in the 15 × 10 × 5 cm acclimatization chamber for 2 min before their behaviour was tested. The arrows represent the direction of the air flow

We defined repellency of a permethrin-treated net as the preference of mosquitoes for an untreated net when given the choice of feeding on a blood source through an untreated net or a permethrin-treated one. We measured the preference with a two-way choice apparatus (Fig 1), where mosquitoes could choose to fly towards and attempt to bite an arm held onto a cage containing either a 30 × 30 cm piece of net with permethrin (270 mg/m^2^) or netting without permethrin (Trek 1 or Trek, Katadyn France, Grenoble, France). A ventilator guided the odours (at a speed of about 20 cm/s) from the two cages into a central cage, into which the mosquitoes had been placed. The side of the insecticide was alternated among tests so that any side preference was avoided. Moreover, the potential bias for one of the sides of our setup was controlled by testing shortly before our experiment that there was no preference for either side when uninfected mosquitoes were given the choice of two untreated net. In eight replicates (each with 10 uninfected mosquitoes aged 20 to 22 days) a repeated G-test of goodness-of-fit [28] showed strong support for no preference [total-G = 1.48, *df* = 8, *P* = 0.99, pooled-G = 0.385, *df* = 1, *P* = 0.53, with no heterogeneity among the replicates (*G* = 1.1, *df* = 7, *P* = 0.99); Additional file 1: Table S3].

Twenty to twenty-two days after the blood meal we tested 90 uninfected mosquitoes and 80 infected ones in groups of 10. For each replicate, we placed the 10 mosquitoes into the central acclimation chamber and KT put his arms as attractants onto the nets of the two small cages. After letting the mosquitoes acclimatize for 2 min we opened the chamber and gave the mosquitoes 8 min to make a choice. We recorded the number of mosquitoes in each cage and put the mosquitoes into a freezer (-20 °C) until further analysis.

### *Malaria infection*

DNA was extracted with DNAzol (MRC inc., Cinncinati, OH, USA) according to the manufacturer’s instructions modified by Rider et al. [29]. The head and thorax of mosquitoes were smashed with a pellet in 200 μl of DNAzol. The solution was incubated for 20 min at 55 °C and then centrifuged at 20,000× *g* for 10 min. One hundred and seventy microliters of the supernatant were transferred to a tube together with 3 μl of PolyAcryl Carrier (MRC inc.) and 100 μl of cold (-20 °C) 100% ethanol. After centrifuging at 15,000× *g* for 8 min we discarded the ethanol. We then washed the DNA by adding 0.8 ml of cold 75% ethanol and centrifuging the tube for 5 min at 15,000× *g*. Ethanol was again discarded and the pellet of DNA was dried in a Speedvac for 15 min at 45 °C. Finally, the DNA was eluted in 50 μl of autoclaved, deionised water.

Sporozoite infection was confirmed by PCR using a modified protocol established by Rider et al. [29]. PCR amplification was done with the T3000 thermocycler [Analytik Jena AG (formerly Biometra), Jena, Germany] with 3 μl of DNA and 1 μl of a 10 μM solution of each primer, forward (5'-ACG ATG ATA TAG ATC AAA T-3') and reverse (5'-TAC CTA AGC TTC TTG CGT A-3'), which amplify a 111 bp sequence of the merozoite surface protein-1 (*MSP-1*) gene of *Plasmodium berghei* NK65 and ANKA. DNA was amplified during 40 cycles with denaturation at 95 °C, annealing at 54 °C and extension at 72 °C, each for 45 s. Infection was confirmed from the correct band of DNA after electrophoresis on a 1.8% agarose gel stained with the RedSafeTM Nucleic Acid stain (iNtRON Biotechnology, Seongnam-si, South Korea).

### *Mosquito body size*

Wing length was taken as a measure of mosquito body size. Wings were placed onto a glass slide and scanned. We used the software ImageJ to measure the distance from the distal end of the alula to the tip of the wing (vein R3) without the fringes [30, 31].

### *Statistical analysis*

We analysed the proportion of mosquitoes that responded with a generalized linear mixed model (GLMM) with a binomial distribution where the infection status was set as a nominal factor and the replicate as a random factor (Additional file 1: Table S2). Using only the mosquitoes that responded, we analysed the preference with a GLMM, where the choice of mosquitoes was set as the response variable, the infection status as a nominal factor, the wing length and the day of test (i.e. the mosquito’s age) as covariates, and the replicate as a random factor (Additional file 1: Table S1). We excluded exposed but not infected mosquitoes from the analyses, as there were too few (*n* = 7) that responded during the test to enable any meaningful statistical analysis or interpretation. The significance of the effects was determined with the ANOVA function (type II) from the “*car*” package [32]. The analysis was performed with the software R [33] and the Rstudio interface [34]. For the mixed effect models, we used the package “*lme4*” [35].

### *Plasmodium falciparum* and *Anopheles arabiensis*

#### Study site and patients

The study was conducted from the beginning of May to the end of July 2016 near Bagomoyo in the coastal region of Tanzania, where *P. falciparum* is the main parasite causing malaria. All laboratory-based work was done at the Kingani insectary site of the Bagamoyo Research and Training Unit (a branch of the Ifakara Health Institute). Malaria parasites were obtained from 6 to 14 year-old children from villages around Bagomoyo. A first blood screening was done at the schools of the villages. We used malarial rapid diagnosis test (SD BIOLINE Malaria Ag P.f/Pan, Standard Diagnostic, Gyeonggi-do, South Korea) to determine if children were infected or not. Children carrying both *P. falciparum* and another *Plasmodium* species were treated with antimalarial drugs (Coartem©, Novartis, Basel, Switzerland) according to the national guidelines. Blood smears of children infected only with *P. falciparum* were coloured with 10% Giemsa and checked under a microscope (oil film 100× magnification) for the presence of gametocytes. Children that harboured only the asexual form of *P. falciparum* were treated with antimalarial drugs (Coartem©, Novartis) at school. Children that harboured gametocytes were driven together with their parents or their teacher to the laboratory of Kingani, where we took a sample of 3 ml of blood to infect mosquitoes (see below). They were then driven back to their villages and the children received an antimalarial treatment.

### *Mosquitoes*

We used the mosquito *An. arabiensis* from a partially permethrin-resistant colony established in 2006 in Sakamaganga, Kilombero, Tanzania and maintained in an insectary at the Bagamoyo Research and Training Unit. Mosquitoes were maintained at a temperature of 27 ± 2 °C with a relative humidity of 70–80% and 12:12 h of dark:light photocycle. Larvae were reared in groups of 250 mosquitoes in trays containing 1 l of water and given the quantities of Tetramine fish food mentioned above. Adults were maintained in 35 × 35 × 35 cm cages and allowed to feed on a 10% glucose solution. Males were removed from the cages every 24 h to avoid fertilization. Twenty-four hours before the infection, female mosquitoes were starved and placed in groups of 50 into paper cups.

### *Blood preparation and infection of mosquitoes*

Mosquitoes were fed on the blood of 5 children containing gametocytes of the human malaria parasite, *Plasmodium falciparum*, obtained as described above. The blood was prepared according to the procedure described by Bousema et al. [36]. The blood was centrifuged for 2 min at 3000× *g*. The serum was removed and replaced with a European AB serum, so that transmission-blocking factors were avoided [37]. Half of the blood of each child was maintained at 37 °C; the other half was heated to 43 °C for 15 min to kill the malaria parasites, preventing infection of mosquitoes without changing other blood characteristics [38, 39]. Mosquitoes were then allowed to feed in the dark for 45 min from micro-feeders containing 300 μl of infected or heat-killed blood maintained at 37 °C. Twenty-four hours after infection, unfed mosquitoes were removed. The fed ones were placed in groups of 20 to 25 individuals into new cups and maintained in a 2.5 security laboratory at 25–27 °C and 70–80% humidity, with 12:12 h of dark:light photocycle. They were allowed to feed on a piece of cotton soaked in a 10% glucose solution until 24 h before the behavioural tests. The tests were done 14 days after feeding, enabling the malaria parasite to produce sporozoites.

### *Measuring repellency and 24 h mortality*

**Fig. 2:**
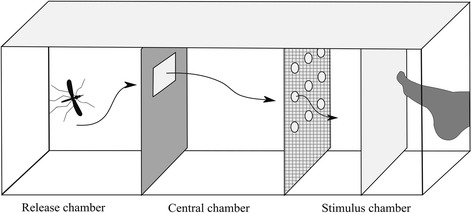
Modified WHO tunnel to test the repellency of ITNs during the experiment with *P. falciparum* infecting *An. arabiensis*. Mosquitoes were placed in the released chamber and allowed to fly in the direction of the stimulus chamber where KT put one of his feet (protected by chiffon with 150 μm mesh). They had to pass through an opening made with cardboard with a rectangular hole of 8 × 12 cm to be considered as responsive, and then could pass through a net with holes to approach KT’s foot if they were not repelled. The arrows represent an example route for mosquitoes to go to the stimulus chamber

In this experiment we assessed whether the attraction of a host to a hungry (unfed) mosquito can overcome the repellency by the permethrin. We measured the repellency of the bednets to infectious or uninfected mosquitoes with a modified WHO tunnel (see Fig. 2). Mosquitoes were placed according to infection status and isolate in groups of 20 into one chamber (the release chamber) of the tunnel. KT placed his foot into the chamber on the opposite side (the stimulus chamber) to attract mosquitoes. His foot was protected from being bitten by a cloth with 150 μm mesh, which enables volatiles but not mosquitoes to pass through. The central chamber was separated from the stimulus chamber by a commercially available permethrin-treated mosquito net (Olyset net, Sumitomo Chemical Co. Ltd., Tokyo, Japan) or by an untreated mosquito net. The nets contained 7 holes (0.8 cm diameter), so that mosquitoes could pass through. The tunnel was covered with black tissue, for *An. arabiensis* prefers to bite during the night. We left mosquitoes in the tunnel for 20 min and then recorded the number of mosquitoes in each part of the tunnel. They were considered as unresponsive, if they remained in the release chamber; responsive but repelled if they were found in the central chamber; and responsive and not repelled if they were found behind the net in the stimulus chamber. After the test, mosquitoes were placed in paper cups for 24 h with access to 10% glucose solution and their 24 h mortality was recorded. They were then placed into Eppendorf tubes containing silica gel for transport to the University of Neuchâtel for further analysis.

### *Malaria infection*

Sporozoite-infection of mosquitoes was confirmed by PCR using the protocol established by Padley et al. [40]. DNA extraction of the head and thorax of mosquitoes was performed with DNAzol according to the procedure described above.

PCR was performed with the T3000 thermocycler (Analytik Jena AG). Forward (5'-AAC AGA CGG GTA GTC ATG ATT GAG-3') and reverse primers (5'-GTA TCT GAT CGT CTT CAC TCC C-3') amplified a 276 bp sequence of *Plasmodium falciparum*. PCR was done on 2 μl of DNA with 240 nM of each primer for a final volume of 25 μl. DNA was amplified during 40 cycles with denaturation at 95 °C, annealing at 60 °C and extension at 72 °C, each for 45 s. Infection was confirmed from the correct band of DNA by electrophoresis on a 1.8% agarose gel stained with the RedSafeTM Nucleic Acid stain (iNtRON Biotechnology).

PCR amplifications were also done on the abdomens of mosquitoes that were exposed to infected blood in order to detect oocyst infection. We followed the same procedure for the PCR and the DNA extraction as for sporozoite-infection, except that we added only 150 μl of DNAzol for each abdomen.

### *Mosquito body size*

The wing length was measured as the distance from the distal end of the alula to the tip of the wing (vein R3) without the fringes.

### *Statistical analysis*

We estimated repellency in two ways: (i) as the proportion of mosquitoes that did not respond to the human cue (Additional file 2: Table S5), i.e. that stayed inside the releases chamber (Fig. 2); and (ii) as the proportion of responding mosquitoes that did not pass the net to move from the central to the stimulus chamber (Additional file 2: Table S4). The former is an expression of spatial repellency, the latter could be due to a combination of spatial repellency and contact irritancy. We analysed the likelihood to respond to human cues with a GLMM where the type of net and the infection status [unexposed (fed on blood containing heat-killed parasites); exposed but not infected; or infectious] were nominal factors, wing length was a covariate and the isolate (the child from whom the parasite had been obtained) and the replicate (the group of 20 mosquitoes tested simultaneously) were random factors. We analysed the likelihood that responding mosquitoes passed through the net with a GLMM that included the type of net and the infection status as nominal factors, wing length as a covariate, and the isolate and the replicate as random factors. Mosquitoes that were infected, but harboured only oocysts, were removed from the analyses. We further analysed to variations of this basic model. First, as the true infection status of ‘exposed but uninfected’ mosquitoes was not clear, we left them out in a second analysis; this did not change the qualitative results. Secondly, we compared the repellency of the treated and untreated nets separately.

Mosquito mortality 24 h after the test of repellency was also analysed with a GLMM (Additional file 2: Table S4). The model included whether mosquitoes died or not as the response variable, and the same factors and covariate as the analysis for repellency. We also compared the mortality of each net individually.

All analyses were performed with the software R [33] and the Rstudio© interface (version 1.0.143) [34]. For the mixed effect models (which were done with the package “*lme4*” [35]), the statistical significance of each factor and covariate was determined with a type 3 ANOVA from the package “*car*” [32].

## Results

### *Plasmodium berghei* and *Anopheles gambiae*

Of the 80 mosquitoes exposed to gametocytic mice, 35 did not harbour sporozoites and were therefore excluded from the analysis. The percentage of mosquitoes responding to human cues was 55.6% (95% confidence interval, CI: 45.3–65.4%) if they were uninfected and 66.7% (95% CI: 52.1–78.6%) if they harboured sporozoites. This difference was not statistically significant (*χ*^2^ = 1.52, *df* = 1, *P* = 0.22).

**Fig. 3:**
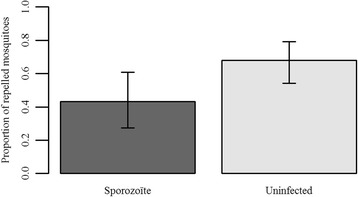
Proportion of repelled mosquitoes as a function of their infection status. Mosquitoes were considered as repelled if they were found in the cage without the permethrin-treated net. The bars represent the 95% confidence interval

Including only the mosquitoes that responded let us analyse the choice of 80 mosquitoes (30 infected and 50 uninfected) when facing a permethrin-treated or a control net. While 68% (95% CI: 54.2–79.2%) of the uninfected mosquitoes avoided the cage with the insecticide, only 43.3% (95% CI: 27.4–60.8%) of the infected ones did (*χ*^2^ = 4.11, *df* = 1, *P* = 0.043) (Fig. 3). Neither the mosquito’s wing length (*χ*^2^ = 0.46, *df* = 1, *P* = 0.5) nor its age (*χ*^2^ = 0.06, *df* = 1, *P* = 0.81) affected the choice of mosquitoes. There was also no effect of the interaction between mosquito’s age and their infection status (*χ*^2^ = 0.04, *df* = 1, *P* = 0.85).

### *Plasmodium falciparum* and *Anopheles arabiensis*

Of the mosquitoes that survived up to 14 days after blood-feeding, we selected haphazardly 80 mosquitoes from each of 3 isolates, 160 from a fourth one and 360 from a fifth (these numbers reflect the greater survival for the latter two isolates). We tested 50 mosquitoes that had fed on blood with heat-killed parasites; none of them were infected. Of the mosquitoes that had fed on infectious blood, 29.0% harboured sporozoites (half of these also harboured oocysts; in our analyses we did not distinguish between the mosquitoes with or without oocysts, both groups were considered as infectious). A further 6.3% harboured oocysts, but had no sporozoites; we removed these from the analysis.

The percentage of mosquitoes that responded to host cues (that is, that moved out of the release chamber) was 83.7% (95% CI: 79.6–87.1%) for the unexposed ones (those fed on blood containing heat-killed parasites), 79.3% (95% CI: 73.8–83.9%) for exposed-but-uninfected mosquitoes and 89.1% (95% CI: 81.9–93.7%) for the infectious ones, (*χ*^2^ = 7.4, *df* = 2, *P* = 0.02). The difference of the percentage of responders between the untreated and treated nets was less than 2% for each infection status (main effect of net: *χ*^2^ = 0.01, *df* = 1, *P* = 0.92; interaction between infections status and net: *χ*^2^ = 0.13, *df* = 2, *P* = 0.94). Wing length had no effect on the proportion of responders (*χ*^2^ = 1.98, *df* = 2, *P* = 0.16).

The analysis comparing the proportion of mosquitoes passing the net into the stimulus chamber included 611 responding mosquitoes, with 286 mosquitoes (151 not exposed, 89 exposed-but-uninfected and 46 infectious) that were tested on an untreated net and 325 (167 not exposed, 106 exposed-but-uninfected and 52 infectious) mosquitoes that were tested on the permethrin-treated Olyset net. About of the quarter of the responding mosquitoes remained in the central chamber, if they were tested with an untreated net. This proportion was not affected by infection status: unexposed: 25.2% (95% CI: 18.9–32.9%.); exposed-but-uninfected: 25.8% (95% CI: 17.9–35.8%); infectious: 26.1% (95% CI: 15.6–40.3%) (*χ*^2^ = 0.05, *df* = 2, *P* = 0.97). When tested on an Olyset net, in average 40.6% of mosquitoes were repelled, with the response ranging from 46.7% (95% CI: 39.3–54.3%) of the unexposed mosquitoes down to 23.1% (95% CI: 13.7–36.1%) of the infectious mosquitoes (*χ*^2^ = 7.67, *df* = 2, *P* = 0.02). Pairwise comparisons showed that unexposed mosquitoes were repelled more than sporozoite-infected ones (*z* = 2.71, *P* = 0.007), and that exposed-but-uninfected mosquitoes were not significantly different from either unexposed (*z* = 1.23, *P* = 0.22) or infectious ones (*z* = 1.74, *P* = 0.08).

The full analysis, pooling the data with the two nets, confirmed that the mosquitoes were less likely to pass through the Olyset than the untreated net (*χ*^2^ = 16.06, *df* = 1, *P* < 0.001), and that the effect of the net depended on the infection status. While the interaction between net type and infection was not quite significant when we compare all three infection groups (*χ*^2^ = 4.66, *df* = 2, *P* = 0.098), it was significant when we omitted the exposed-but-uninfected mosquitoes (*χ*^2^ = 4.44, *df* = 1, *P* = 0.035). Wing length did not affect the behavioural response in either of these analyses (three infection groups: *χ*^2^ = 0.02, *df* = 1, *P* = 0.90; unexposed *vs* infectious *χ*^2^ = 0.45, *df* = 1, *P* = 0.51).

**Fig. 4:**
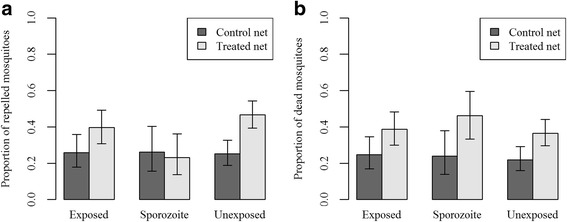
**a** Proportion of mosquitoes found in the central cage (and therefore responded to the odour but were repelled by the net) for the two types of net [Olyset permethrin-treated net (light grey) or control untreated net (dark grey)] and for the three infection statuses. **b** Proportion of mosquitoes that died within 24 h after the behavioural test, as a function of the type of net and infection status. These proportions represent the overall mortality, combining the mortality of the mosquitoes considered as repelled and the one considered as unrepelled. For both graphs the bars represent the 95% confidence interval

Overall, uninfected mosquitoes were more repelled by the Olyset than the untreated net, but infectious mosquitoes were just as likely to pass through the Olyset as the untreated net (Fig. 4a).

Twenty-four hours after the test with the untreated net 23.1% of mosquitoes had died, independently of their infection status: unexposed 21.9% (95% CI: 16–29.1%); exposed-but-uninfected: 24.7% (95% CI: 16.9–34.6%); infectious: 23.9% (95% CI: 13.9–37.9%) (*χ*^2^ = 0.55, *df* = 2, *P* = 0.76). After the test with a permethrin-treated net, 38.8% of mosquitoes had died, again independently of their infection status unexposed: 36.5% (95% CI: 29.6–44.1%); exposed-but-uninfected: 38.7% (95% CI: 30–48.2%); infectious: 46.2% (95% CI: 33.3–59.5%) (*χ*^2^ = 1.45, *df* = 2, *P* = 0.48).

In the analysis pooling the two types of net, we found that the Olyset net increased the risk of dying by a factor of 1.7 (*χ*^2^ = 15.05, *df* = 1, *P* < 0.001, Fig. 4b) and we confirmed that there was no significant effect of the infection status (*χ*^2^ = 1.48, *df* = 2, *P* = 0.48) and that neither the wing length (*χ*^2^ = 0.0002, *df* = 1, *P* = 0.99) nor the interaction between the type of net and the infection status (*χ*^2^ = 0.46, *df* = 2, *P* = 0.79) affected the mosquito’s mortality.

## Discussion

Our results with two experimental systems show that infection by malaria affects the behavioural response of mosquitoes to insecticides. In particular, in both systems, infection by sporozoites negated the repellency of permethrin, therefore decreasing the personal protection offered by an ITN.

The decrease of repellency was not only observed with two combinations of malaria and mosquito species, but also with two ways of measuring the repellent effect of an insecticide. In the first experiment, when given the choice between flying into (and biting in) a chamber containing either an untreated or a treated net, uninfected mosquitoes preferred to fly into the chamber that was not protected by permethrin (showing that they were repelled by the insecticide), while infectious mosquitoes showed no preference. In the second, mosquitoes were not given the choice of an insecticide-free chamber, but we measured the likelihood that mosquitoes would pass an untreated or a treated net to get to their blood meal. Again, uninfected mosquitoes were repelled, and thus less likely to fly through a treated bednet than a treated one to reach their source of blood, while infectious mosquitoes responded similarly to treated and untreated nets.

Both of these aspects of repellency could be a consequence of spatial repellency (which impede mosquitoes from approaching treated nets) or contact irritancy (which makes mosquitoes fly away once they contact a treated net). However, our second experiment gives some evidence that spatial repellency is not an important factor. If it were important, we would have expected that fewer mosquitoes move towards a treated net (and thus stay in the release chamber) than towards an untreated net. The proportions, however, did not differ. This corroborated the patterns observed in several other studies that found no spatial repellency, but strong irritancy for permethrin (reviewed in [7]).

The proportion of mosquitoes remaining in the release chamber did, however, depend on their infection: sporozoite-infected mosquitoes were more likely to move out of the chamber and towards the human cues than uninfected or exposed-but-uninfected mosquitoes. This is in line with previously observed changes of mosquitoes’ biting behaviour induced by malaria, in particular that sporozoite-infection increases the host attraction [41] and the motivation and persistence of mosquitoes to obtain a blood meal [19–23] by enhancing the responsiveness of mosquito’s olfactory receptors [41].

The possible mechanism of the decreased repellency of infectious mosquitoes by permethrin may be based on this behavioural manipulation: infectious mosquitoes could be more motivated to pass the permethrin-treated net to obtain their blood meal than uninfected ones. Alternatively, the chronic infection by malaria may simply damage in some way the receptors (or their integrative pathways) of the mosquitoes, analogously to the effect of a previous exposure to an insecticide in the odour receptors of *Aedes aegypti* [42]. Thus the mosquitoes would be less aware of the insecticide’s odour or irritancy, and therefore less repelled by it. Indeed, malaria infection reduces the levels of several proteins in the head of mosquitoes, including a synaptic and a neural signaling protein, though the functions of these proteins are not known [43].

Our observations on mortality are a bit less straightforward than those on behavior. As expected, the permethrin-treated net killed more mosquitoes than the untreated net. However, although the sporozoites increased the mortality from about 37% to about 46%, this difference was far from statistically significant. This contrasts our expectation that the increased contact of infectious mosquitoes with the insecticide would increase the likelihood that they are killed. A likely explanation for the lack of statistical significance is simply a lack of power. If (as the numbers of our experiment indicate) about 20% fewer mosquitoes are repelled and these are 10% more likely to be killed, we would need a large sample size to detect the combined effect as a statistically significant result. Alternatively, infectious mosquitoes may pass through the net so quickly that their exposure is too short to kill them. It would be important to know which of these (or any other) explanation is correct, for community protection and the rate of the evolution of insecticide resistance both depend mainly on the insecticidal property of ITNs [3]. Furthermore, to fully determine the level of community protection offer by ITNs it would be important to take in account the weaker attraction or motivation of oocyst-infected mosquitoes [23, 41]. Indeed, it might increase the overall number of infectious mosquitoes, for oocyst infection might decrease the mosquito’s risk of dying due to ITNs or host defence behaviours [23, 44].

A caveat of our study is that both experiments were done in laboratory conditions, which can differ considerably from natural situations. In particular, laboratory infections might differ from natural infection and thus might influence the density of the sporozoites in the mosquitoes’ salivary glands [45–47], which in turn can influence the malaria-induced change of behaviour [24]. Nevertheless, we suggest that malaria infection in mosquitoes can decrease the repellency of insecticides and may reduce the personal protection at the stage when the protection is most relevant.

## Conclusions

Insecticide-based tools, like ITNs and IRS, have proven their efficacy against malaria [2, 48] and remain the most cost-effective tools for malaria control [1]. However, to fully understand and predict the impact of ITNs on the epidemiology of malaria, we need to know how malaria affects the personal and community protection offered by the nets. Our main finding is that the malaria infection, at least in the laboratory, reduces the personal protection offered by insecticides. We suggest that further studies should investigate whether our laboratory results are consistent in the field and go on to investigate whether the less effective personal protection observed here can be balanced by increased community protection.

## Additional files

Additional file 1: **Table S1.** *P. berghei* - *An. gambiae* dataset. Data from the laboratory experiment with *An. gambiae* and *P.berghei* used for statistical analysis of repellency. **Table S2.** *P. berghei* - *An. gambiae* dataset. Data from the laboratory experiment with *An. gambiae* and *P.berghei* used for statistical analysis of the proportion of responding mosquitoes. **Table S3.** *P. berghei* - *An. gambiae* dataset. Data from the laboratory experiment with *An. gambiae* and *P. berghei* used for statistical analysis of the side preference. (XLSX 16 kb)

Additional file 2: **Table S4.** *P. falciparum* - *An. arabiensis* dataset. Data from the experiment with *An. arabiensis* and *P. falciparum* used for statistical analysis of repellency. **Table S5.** *P. falciparum* - *An. arabiensis* dataset. Data from the experiment with *An. arabiensis* and *P. falciparum* used for statistical analysis of the proportion of responding mosquitoes. (XLSX 50 kb)

## Abbreviations

CI: Confidence interval
GLMM: Generalized Linear Mixed Model
IRS: Indoor residual spraying
ITN: Insecticide-treated net
